# Two-Dimensional Capillary Electrophoresis with On-Line Sample Preparation and Cyclodextrin Separation Environment for Direct Determination of Serotonin in Human Urine

**DOI:** 10.3390/molecules22101668

**Published:** 2017-10-07

**Authors:** Juraj Piešťanský, Katarína Maráková, Peter Mikuš

**Affiliations:** 1 Department of Pharmaceutical Analysis and Nuclear Pharmacy, Faculty of Pharmacy, Comenius University in Bratislava, Odbojárov 10, SK-832 32 Bratislava, Slovakia; piestansky@fpharm.uniba.sk (J.P.); marakova@fpharm.uniba.sk (K.M.); 2 Toxicological and Antidoping center, Faculty of Pharmacy, Comenius University in Bratislava, Odbojárov 10, SK-832 32 Bratislava, Slovakia

**Keywords:** serotonin, bioanalysis, capillary electrophoresis, column coupling, cyclodextrin, biomarker, on-line sample preparation, human urine

## Abstract

An advanced two-dimensional capillary electrophoresis method, based on on-line combination of capillary isotachophoresis and capillary zone electrophoresis with cyclodextrin additive in background electrolyte, was developed for effective determination of serotonin in human urine. Hydrodynamically closed separation system and large bore capillaries (300–800 µm) were chosen for the possibility to enhance the sample load capacity, and, by that, to decrease limit of detection. Isotachophoresis served for the sample preseparation, defined elimination of sample matrix constituents (sample clean up), and preconcentration of the analyte. Cyclodextrin separation environment enhanced separation selectivity of capillary zone electrophoresis. In this way, serotonin could be successfully separated from the rest of the sample matrix constituents migrating in capillary zone electrophoresis step so that human urine could be directly (i.e., without any external sample preparation) injected into the analyzer. The proposed method was successfully validated, showing favorable parameters of sensitivity (limit of detection for serotonin was 2.32 ng·mL^−1^), linearity (regression coefficient higher than 0.99), precision (repeatability of the migration time and peak area were in the range of 0.02–1.17% and 5.25–7.88%, respectively), and recovery (ranging in the interval of 90.0–93.6%). The developed method was applied for the assay of the human urine samples obtained from healthy volunteers. The determined concentrations of serotonin in such samples were in the range of 12.4–491.2 ng·mL^−1^ that was in good agreement with literature data. This advanced method represents a highly effective, reliable, and low-cost alternative for the routine determination of serotonin as a biomarker in human urine.

## 1. Introduction

Serotonin (5-hydroxytryptamine, 5-HT) is a biogenic amine synthetized from amino acid l-tryptophan in enterochromaffin cells of intestinal mucosa. This molecule is predominantly located in the gastrointestinal tract (GIT), blood platelets, and central nervous system (CNS). The 5-HT functions as a neurotransmitter in the gut and in the brain, as a paracrine messenger in the gut, and as a hormone in the periphery [1,2]. Peripheral 5-HT is a potent immune modulator and affects various immune cells. Alterations in 5-HT signaling have been described in inflammatory conditions of gut (e.g., inflammatory bowel disease, IBD), allergic airway inflammation or rheumatoid arthritis [3]. Hence, 5-HT represents a very attractive molecule for its consideration as a biomarker. However, some of its functions are still the subject of investigation that would not be possible without the development of accurate, precise, and sensitive analytical methods implemented in bioanalysis [4].

Capillary electrophoresis (CE) represents an attractive and effective tool for the analysis of ions or small ionogenic compounds, which has proved competitive/complementary to high-performance liquid chromatography (HPLC) [5,6]. This separation method is characterized by many advantages—such as high separation efficiency, versatility, flexibility, use of aqueous separation systems, and low consumption of electrolytes and sample. On the other hand, CE also has some drawbacks, which limits its application in routine analytical laboratories (e.g., relatively difficult optimization of separation system, lower reproducibility of measurements in comparison to HPLC, and low sample load capacity increasing demands for the preconcentration of the sample).

A unique role of multidimensional CE separation techniques in the solution of various bioanalytical problems including the analysis of multicomponent matrices is demonstrated in some recent excellent reviews [7,8,9]. These papers summarize and discuss trends in the column-coupling strategies (types of interfaces), 2D separation in a single capillary, off-line 2D systems, microfluidic devices, and advances in the hyphenations of 2D CE with the selective and sensitive detection techniques, such as laser-induced fluorescence or mass spectrometry. A growing interest in the multidimensional CE separation methods is supported by the current papers associated with the heart-cut analysis of proteins [10,11,12], drug degradation products [13], neutral and cationic analytes [14], or 2D CE analysis of selected amino acids [15]. These papers introduce innovative interfaces in the CE column-coupling arrangement based on a fully electric isolated mechanical valve [12], 4-port valve [13], or demonstrate effective strategies for zero-dead volume microfluidic interface [15]. An importance of the coupling of two small internal diameter capillaries in ultrasensitive 2D CE approach of proteins was clearly illustrated on the capillary isoelectric focusing (CIEF) and CZE combination [11].

An advanced column-coupling arrangement employing wide-bore capillaries in a modular hydrodynamically closed separation system can be a very effective approach offering additional benefits to the conventional single column CE technique and overcoming some of its disadvantages [16,17,18]. Two separate stages can provide a sample preparation and analytical separation of an original (i.e., unpretreated) sample in one compact experiment. The column-coupling arrangement involves combination of two different electrophoretic separation mechanisms (isotachophoresis, ITP, and capillary zone electrophoresis, CZE), and, by that, enhanced orthogonality of the system (enhanced separation selectivity). Further, it offers well-defined elimination of undesirable sample matrix constituents during an ITP preseparation, and substantial enhancement of the sample load capacity due to employing a large bore ITP capillary (usually 800 µm), easily applicable in the hydrodynamically closed separation system. Moreover, a column-coupled system, integrating the sample preparation and analysis into one run, can provide an improved precision of the analyses due to a minimization of external sample manipulation.

Several papers deal with the CE analysis of 5-HT in different biological sample matrices, such as urine [19,20,21,22,23,24,25,26,27], plasma, serum [22,28], blood [22,29], brain tissue [30,31], bowel content [32], neurons [33,34], and commercially available food supplements [35,36]. Most of the cited CE methods were accompanied with either external (liquid-liquid extraction—LLE, solid phase extraction—SPE) or on-line (various stacking methods, e.g., field-amplified sample injection, dynamic pH junction, transient isotachophoresis) sample pretreatment in order to decrease limit of detection (LOD). Moreover, a combination of CE separation with highly sensitive and selective detection (e.g., laser induced fluorescence—LIF, electrochemical/amperometric detection, mass spectrometry—MS) provided additional enhancement of the sensitivity and selectivity. Obtained LOD values of serotonin were, or ranged in the intervals of 1.04–4.41 ng·mL^−1^ (UV), 3.5–35 pg·mL^−1^ (LIF), and 5.52 ng·mL^−1^ (amperometric detection) in human urine; 176 ng·mL^−1^ (UV) and 546 ng·mL^−1^ (electrochemical detection) in food supplements; 17.62 ng·mL^−1^ (UV) in blood; 52.87 pg·mL^−1^ (amperometric detection) and 70.47 pg·mL^−1^ (LIF) in brain tissue; 88.11 pg·mL^−1^ (electrochemical detection) in serum; and 1.76 ng·mL^−1^ (LIF) and 6.17 ng·mL^−1^ (mass spectrometry—MS) in neurons. In this field, however, a column-coupled CE technique has not been considered so far.

The aim of this work is to develop and apply an advanced 2D CE method employing column-coupled experimental arrangement for the analysis of 5-HT in human urine. It is expected, considering our recent works (see e.g., [37]), that such method would effectively analyze directly injected, unpretreated (except for a proper dilution) complex biological samples in one run due to on-line integrating ITP preseparation and CZE analysis steps. However, as this is a highly specialized field and the 2D CE modular equipment in a hydrodynamically closed separation system is not conventional (is rarely used), the experiments need highly skilled operators with a deep knowledge in ITP methodology, creating separation conditions suitable for wide bore capillaries, and performing heart-cut experiments. An optimization of the ITP conditions for a proper preconcentration and sample clean-up is included in the method development along with an optimization of the integrated CZE step. Here, various additives such as organic modifiers and complexing agents (cyclodextrins) are tested to achieve a complete separation of 5-HT from a rest of interfering sample matrix constituents. The optimized 2D CE method with evaluated performance parameters should be suitable for the determination of 5-HT as a biomarker in human urine samples.

## 2. Results and Discussion

### 2.1. Development of ITP–CZE–UV Method

Separation conditions of the 2D CE method, based on the combination of column-coupled ITP and CZE steps, were optimized to achieve an acceptable compatibility and performance of both integrated steps and complete separation of trace 5-HT from the urine sample matrices containing a big amount of major as well as minor interfering matrix constituents.

**Figure 1:**
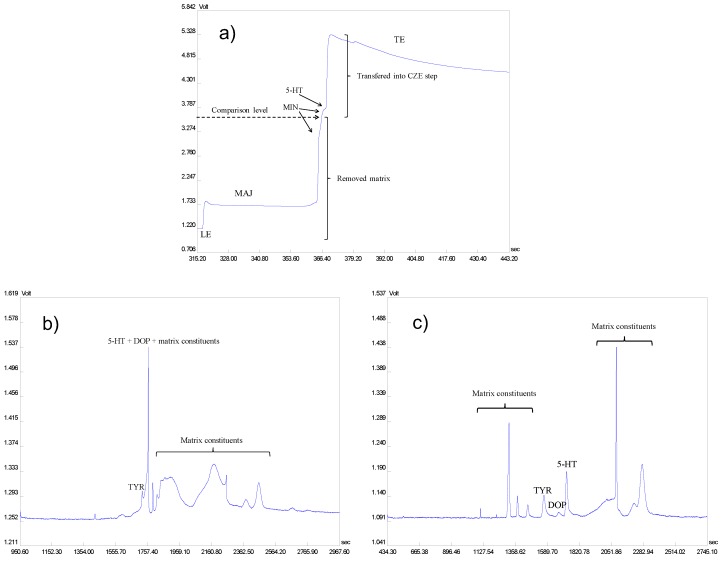
Isotachophoresis–capillary zone electrophoresis–ultraviolet (ITP–CZE–UV) analysis of model urine samples spiked with three biogenic amines—5-hydroxytryptamine (5-HT), dopamine (DOP), and tyramine (TYR). (**a**) Optimized ITP profile. (**b**) CZE profile without additives influencing separation of the analytes, background electrolyte (BGE): 25 mM ε-aminocaproic acid (EACA) + 50 mM acetic acid (HAc) + 0.1% m-HEC. (**c**) Optimized CZE profile with additives influencing separation. The human urine samples were diluted 10 times. The biogenic amines were present at 50 ng·mL^−1^ concentration levels of their hydrochloride salts (41.43 ng·mL^−1^ of 5-HT, 40.39 ng·mL^−1^ of DOP and 39·50 ng·mL^−1^ of TYR base). For other instrumental parameters and optimum ITP and CZE separation conditions see the Section 3.1 and Section 2.1. LE—leading electrolyte, MAJ—major sample matrix constituents, MIN—minor sample matrix constituents, TE—terminating electrolyte.

*ITP optimization.* ITP is a powerful technique for the preconcentration and preseparation of the complex samples [16,38,39]. The composition of leading (LE) and terminating (TE) electrolytes was optimized in order to obtain a proper sample preconcentration and preseparation with the possibility of well-defined selection of the zone of interest (i.e., ITP zone in which 5-HT migrates) from the isotachophoretic sample profile. Such well-defined selection of the zone of interest (based on the fixed-height positions of zones of the compounds migrating in the ITP profile under given ITP conditions) considerably reduces complexity of the sample fraction transferred into the CZE step, and, by that, minimizes the variability of the method to different urine sources. The buffers composed from ammonium acetate (NH_4_Ac) and ammonium formate (NH_4_Fo) with acetic acid (HAc), formic acid (HFo), and 2-(*N*-morpholino)ethanesulfonic acid (MES) were tested as the LE electrolytes. The most effective clean-up of the sample from major matrix constituents was obtained when using buffer composed from NH_4_Ac with MES and lower pH values. Solutions of HAc and HFo or the buffer composed from ε-aminocaproic acid (EACA) with HAc were considered as the TE electrolytes. The buffer composed from EACA with HAc was chosen as it was favorable in term of the lowest effective mobility of terminating ion from among other tested ions. Hence, all sample constituents could be included and effectively preseparated and preconcentrated between the LE and TE zones. In addition, this TE enabled to easily choose a compatible and suitable background electrolyte (BGE) for the CZE separation step. The optimum ITP separation conditions were: LE, 10 mM NH_4_Ac + 20 mM MES + 0.1% m-HEC (methylhydroxyethylcellulose), pH 5.56; TE, 10 mM EACA + 10 mM HAc + 0.1% m-HEC, pH 4.55; resulting ITP profile illustrating an on-line preseparation of the urine sample containing model 5-HT under the optimized conditions is in Figure 1a.

**Figure 2:**
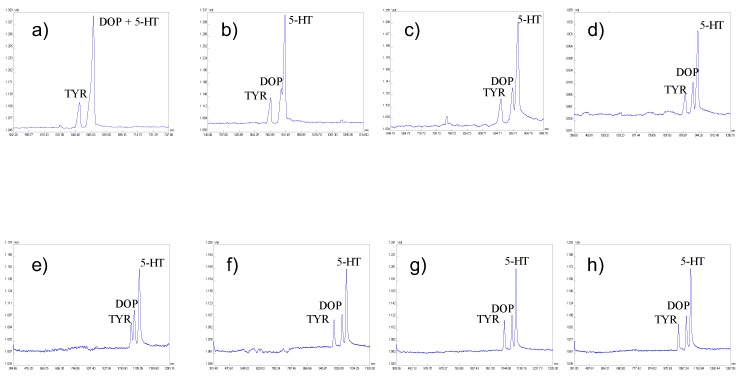
Effect of additives (isopropanol and HP–β–CD) on the CZE separation of mixture of three biogenic amines—5-HT, DOP, and TYR in model water matrix using ITP–CZE–UV experimental arrangement. Concentration levels of the injected hydrochloride salts of 5-HT, DOP, and TYR were 100 ng·mL^−1^ for each analyte that represented a 82.86 ng·mL^−1^ concentration of 5-HT, a 80.77 ng·mL^−1^ concentration of DOP, and a 79.00 ng·mL^−1^ concentration of TYR. Tested BGE compositions were following: 25 mM EACA + 50 mM HAc + 0.1% m-HEC (**a**) -, (**b**) + 10% isopropanol, (**c**) + 20% isopropanol, (**d**) + 30% isopropanol, (**e**) + 10 mM HP–β–CD, (**f**) + 10 mM HP–β–CD + 10% isopropanol, (**g**) + 10 mM HP–β–CD + 15% isopropanol, (**h**) + 10 mM HP–β–CD + 20% isopropanol. A 90 mm length of the CZE separation capillary was employed. For other instrumental parameters and optimum ITP separation conditions, see Section 3.1 and Section 2.1.

*CZE optimization.* Several electrolytes {HFo, HAc, combination of gamma-aminobutyric acid (GABA) with HAc or EACA with HAc} with different composition, concentration, and pH values were tested in order to choose optimum CZE separation conditions for the isotachophoretically pretreated urine sample fraction containing 5-HT and a rest of the sample matrix constituents. Although BGE composed from EACA with HAc provided best separation from among the other tested BGEs, it has still not been able to completely separate 5-HT from the residual matrix, as seen in Figure 1b. A major problem was associated with another biogenic amine, namely dopamine (DOP), that had a very similar migration behavior to 5-HT and created a mixed electrophoretic zone. Therefore, organic modifiers (methanol, isopropanol) as well as complexing agents {carboxyethyl-β-cyclodextrin—CE–β–CD; (2-hydroxypropyl)-β-cyclodextrin—HP–β–CD} were tested as additives to the BGE for improving the separation [37,40,41]. The optimization process of CZE step of the ITP–CZE–UV combination, showing separation of a model mixture of three biogenic amines, namely 5-HT, DOP, and tyramine (TYR), is illustrated in Figure 2.

An addition of methanol had a negligible effect on the separation of DOP and 5-HT peaks from each other. On the other hand, isopropanol was much more effective in this case. Although higher concentrations of isopropanol improved the separations of 5-HT and DOP, a baseline resolution could not be achieved. Moreover, the analysis time was prolonged with increased isopropanol concentration. Implementation of negatively charged CE–β–CD was associated with a shift of the analyzed compounds to higher migration times and still did not achieve acceptable resolution between 5-HT and DOP. Promising results were obtained with the use of HP–β–CD that provided an acceptable separation between 5-HT and DOP. Moreover, when using a proper combination/mixture of HP–β–CD and isopropanol, the baseline resolution of all the tested biogenic amines could be reached as an additional benefit. This is suitable not only for a possibility to determine these three biogenic amines as potential biomarkers in various practical situations, but also for higher robustness of the ITP–CZE–UV method required in bioanalysis. Hence, the optimum CZE separation conditions were: BGE, 25 mM EACA + 50 mM HAc + 10 mM HP–β–CD + 20% isopropanol + 0.1% m-HEC, pH 4.62.

The optimized ITP–CZE–UV method was finally tested by using human urine matrices spiked with 5-HT, DOP, and TYR. Resulting CZE profile, shown in Figure 1c, clearly demonstrated an importance of the optimization process for the baseline separation of 5-HT from the urine matrix constituents including biogenic amines such as DOP and TYR (compare traces a) and c) in Figure 1).

### 2.2. Evaluation of Performance Parameters of ITP–CZE–UV Method

The optimized ITP–CZE–UV method was validated according to the US Food and Drug Administration (FDA) guidance for bioanalytical method validation [42]. During the validation process, the following parameters were evaluated: linearity, selectivity, limit of detection (LOD), lower limit of quantification (LLOQ), accuracy, precision, and stability. Detail overview of the most important validation and operation parameters is summarized in Table 1, Table 2 and Table 3.

The parameters of calibration lines obtained for the samples prepared in water and human urine model matrices (Table 1) were calculated with the use of Microsoft Excel 2007 (Microsoft Corporation, Redmond, WA, USA). The calibration curves for 5-HT were linear (determination coefficient was higher than 0.99) in a wide concentration range (pronouncedly exceeding one decadic order).

Limit of detection (LOD) and lower limit of quantification (LLOQ) were defined as the concentrations of the analyte corresponding to the signal-to-noise ratios (S/N) 3:1 and 5:1, respectively. They were calculated from the standard electropherograms obtained under the optimized working conditions. Favorable LOD and LLOQ values, ranging in lower ng·mL^−1^ levels, were obtained (Table 1). Such concentration levels, demanded in real clinical applications, highlighted usefulness of the method in practice.

Selectivity, as an ability of the method to differentiate and quantify 5-HT in the presence of other components in the sample with variable matrix, was proved by the analyses of three individual blank human urine samples spiked with 5-HT at the LLOQ concentration level. No interference of the 5-HT peak with other urine matrix constituents was observed. Moreover, similar positive results on the selectivity were obtained also when additionally spiking these urine samples with other structurally related biogenic amines, DOP and TYR. These results clearly demonstrated that, due to both the well-defined ITP selection of the zone of interest and the fine CZE separation of such sample fraction in cyclodextrin environment, the different urine sources did not have any significant influence on the variability of the method. Therefore, the developed method can be considered to be selective enough, and, by that, useful for real complex matrices.

Accuracy and precision of the method were evaluated by analyzing quality control (QC) samples prepared at three concentration levels (low, medium, high), see Table 2.

A within-run precision, defined as an assessment of precision during a single analytical run, was evaluated from repeated analyses of the QC samples in one day. A between-run precision, defined as an assessment of precision over time, was evaluated from repeated analysis of the same QC samples performed twice per day over 5 days (n = 10). The relative errors (RE), characterizing accuracy of the method, ranged in the intervals of 6.2–11.1% and 9.5–11.5%, corresponding with the within-run and between-run experiments, respectively. The RSD values, characterizing precision of the method, ranged in the intervals of 2.6–10.1% and 5.7–12.5%, corresponding with the within-run and between-run experiments, respectively. All these values, being below a critical value of 15%, clearly confirmed acceptable precision and accuracy of the ITP–CZE–UV method, and, by that, its suitability for the practical use.

Recovery was tested as a ratio of the analytical signals (peak areas of 5-HT) obtained from the analysis of spiked biological matrix (10 times diluted human urine) and standard (water) matrix with the same analyte concentration; see Table 3. The values ranging in the interval of 90.0–93.6% represented an acceptable recovery of the method and its application suitability for human urine matrices.

The stability of 5-HT in the biological matrices (10 times diluted human urine) was investigated employing the QC samples. Two types of the stability studies were carried out: (i) short-term stability (samples stored 24 hours at room temperature and then measured and compared with the results of freshly prepared QC samples), (ii) freeze and thaw stability (three freeze-thaw cycles based on a freezing and subsequent thawing at room temperature). The obtained results, presented in Table 3, clearly indicated the adequate short-term and freeze and thaw stabilities of 5-HT in human urine matrices with no significant differences observed between nominal and found concentration (peak area) of 5-HT, that is, not exceeding a relative error of 9%. Hence, the proposed method along with the standard sample handling is suitable for its implementation in routine clinical laboratories.

### 2.3. Method Application

**Figure 3:**
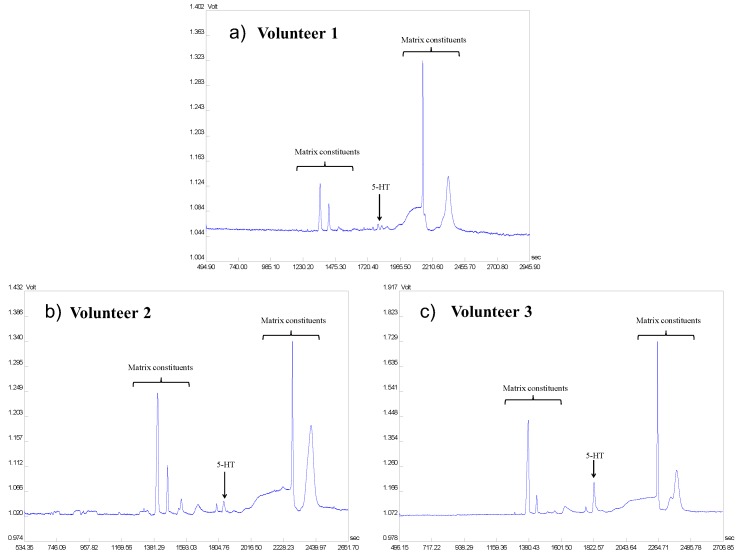
Electrophoretic profiles of urine samples taken from volunteers for screening of 5-HT concentrations. Concentration levels of 5-HT in urine samples obtained from three healthy volunteers (**a**–**c**) ranged in the interval of 12.36–491.23 ng·mL^−1^. The human urine samples were diluted 10 times. For the instrumental parameters and optimum ITP and CZE separation conditions see Section 3.1 and Section 2.1.

The optimized 2D-CE method with favorable performance parameters was applied in practical biomedical analysis such as the screening of 5-HT concentrations in urine of healthy volunteers. Concentration levels of 5-HT in urine samples obtained from three healthy volunteers ranged in the interval of 12.4–491.2 ng·mL^−1^; for corresponding electrophoretic profiles, see Figure 3.

In accordance with these results were the data found in literature ranging from tens to hundreds ng·mL^−1^. For example, the concentration levels of 5-HT found in urine samples were 144.5 ng·mL^−1^ [20], 319 ng·mL^−1^ [21], 52–121 ng·mL^−1^ [24], 72.6–125.3 ng·mL^−1^ [26], and 223.8 ng·mL^−1^ [27]. The successful application of the developed ITP–CZE–UV method supported its practical utilization in routine clinical laboratories aimed to screening of low molecular biomarkers such as biogenic amines including serotonin.

## 3. Materials and Methods

### 3.1. Instrumentation

The ITP–CZE experiments were performed with the use of a modular capillary electrophoresis analyzer EA-102 (Villa Labeco, Spisska Nova Ves, Slovakia), assembled in the column-coupling configuration of the separation units and operated in hydrodynamically (membrane) closed system. The sample was injected by a 30 µL internal sample loop which was a part of the injection valve of the analyzer. The ITP column was provided with an 800 µm I.D. capillary tube made of polytetrafluorethylene (PTFE). Its total length was 90 mm. A contactless conductivity detector was an integral part of the ITP column. The CZE column was provided with a 300 µm I.D. capillary tube made of PTFE of a 160 mm total length. In addition, during the optimization process of BGE composition in the CZE separation step, a 90 mm total length CZE column (with the same characteristics as the 160 mm column) was used. A 0.1% (*w*/*v*) concentration of m-HEC was employed in the separation electrolytes for a dynamic coating of the capillaries to suppress the electroosmotic flow. A photometric detector ECOM (ECOM, Prague, Czech Republic) was connected to an on-column photometric detection cell, integrated in the CZE column, via optical fibers. The detection wavelength was set at 276 nm. To control the separation process and to acquire data from the detectors, ITP Pro software version 1.0 (Villa Labeco, Spisska Nova Ves, Slovakia) was used. The experiments were carried out in a constant current mode at 20 °C (laboratory temperature). The driving currents applied were 300 µA (ITP) and 50 µA (CZE).

### 3.2. Chemicals and Samples

Serotonin (5-hydroxytryptamine, 5-HT), dopamine (DOP), tyramine (TYR), 2-morpholino-ethanesulfonic acid (MES), γ-aminobutyric acid (GABA), ε-aminocaproic acid (EACA), (2-hydroxypropyl)-β-cyclodextrin (HP–β–CD), DS 4.5, were of analytical-grade. Acetic acid (HAc), ammonium acetate (NH_4_Ac), formic acid (HFo), ammonium formate (NH_4_Fo), isopropanol, and methanol were of optimal LC-MS grade. All these chemicals and solvents were purchased from Sigma Aldrich (Steinheim, Germany). Carboxyethyl-β-cyclodextrin (CE–β–CD), DS 3.5, CE purity, was obtained from Cyclolab (Budapest, Hungary). Demineralized water from a Millipore Simplicity 185 (UV) (Millipore, Molsheim, France) water purification system was used for the preparation of electrolyte solutions and samples. All solutions were filtered prior to the use with disposable membrane filters (0.45 μm pore size, Millipore, Molsheim, France) and were stored in the fridge before analysis.

### 3.3. Procedures for Sample and Standard Solution Preparation

#### 3.3.1. Standard Solutions, Calibration Solutions, and Quality Control (QC) Samples

The stock solution of 5-HT reference substance was prepared by dissolving 10 mg of the powder in 10 mL of demineralized water. Working solutions of 5-HT were made by a proper dilution of the stock solution with demineralized water or by spiking human urine with the stock solution of 5-HT.

The concentration levels of 5-HT in the injected calibration solutions, prepared in demineralized water and 10-fold diluted blank urine, were in the range of 2.07–82.86 ng·mL^−1^ (2.07, 4.14, 8.29, 16.57, 41.43, 57.99, 82.86) and 4.14–82.86 ng·mL^−1^ (4.14, 8.29, 16.57, 41.43, 57.99, 82.86).

Quality control samples (QCs) were prepared at three concentration levels—4.14, 24.86 and 66.28 ng·mL^−1^—of 5-HT in 10-fold diluted blank human urine. The prepared QCs correspond to low, medium and high QC concentration levels. Each QCs concentration point was measured five times.

#### 3.3.2. Urine Sample Preparation

The first morning urine was taken from healthy adult volunteers. A 10 mL sample was divided into several vials and frozen (−18 °C) immediately after the sampling and kept in the freezer until the use. Prior to the analysis, the sample was melted and equilibrated for 30 min at room temperature. Each sample was centrifuged and 10-times diluted with demineralized water. After the dilution the sample was gently shaken and filtered through disposable membrane filters (0.22 μm pore size, Millipore, Molsheim, France) and immediately injected into the electrophoretic analyzer.

## 4. Conclusions

The developed ITP–CZE–UV method was presented as a powerful tool for the trace analysis of 5-HT in complex biological samples such as human urine. This column-coupled technique represents an advanced analytical approach as it provides sample treatment and analytical separation in one experiment. A favorable sensitivity and LOD (low ng·mL^−1^ concentration levels) was obtained due to the large volume sample injection (30 µL) combined with the ITP preconcentration. A favorable selectivity was a result of combination of the ITP sample preseparation and clean-up (on-line elimination of majority of sample matrix constituents) with the subsequent fine CZE separation. Here, the CZE separation environment, formed by a proper mixture of BGE with organic modifier (isopropanol) and complexing agent (HP–β–CD), allowed not only the complete separation of 5-HT from the (residual isotachophoretically preseparated) sample matrix constituents but also the complete separation of structurally related biogenic amines (5-HT, DOP, TYR) from each other. Such ITP–CZE operating conditions provided considerable simplification and rationalization of the overall analytical procedure. Moreover, minimization of the sample manipulation (i.e., off-line sample treatment) reflected in the favorable precision and accuracy of the analysis. The analytical arrangement employing the on-line coupled ITP–CZE is able to solve problems with a low selectivity and sensitivity of UV detection, and, by that, provide cheap but highly reliable (ITP–CZE–UV) analyses demanded in the routine clinical laboratories. It is believed that this analytical approach could be useful in the field of gastroenterology or neurology, where changes of 5-HT play an important role in human health. In addition, the possibility to separate the mixture of biogenic amines including 5-HT, DOP, and TYR as potential biomarkers, can further spread the application potential of this approach in bioanalysis.

## Figures and Tables

**Table 1:** Calibration and selected separation parameters for serotonin in model water and human urine matrices.

Performance Parameter	Water	Urine
Slope a	32.94	34.79
SD_a_	0.6125	0.5553
Interface b	3.184	66.016
SD_b_	0.2636	2.4500
r^2^	0.9942	0.9957
Linear range (ng·mL^−1^)	2.07–82.86	4.14–82.86
LOD (ng·mL^−1^)	1.24	2.32
LLOQ (ng·mL^−1^)	2.07	4.14
*N*	40,565	29,898
Migration time t_m_ (min)	27.36	28.73
RSD_tm_ (%)	0.03–0.57	0.02–1.17
RSD_area_ (%)	1.04–4.34	5.25–7.88

Separation efficiency (*N*) was calculated according to the equation *N =* 5.545(t_m_/w_1/2_)^2^*.*

**Table 2:** Accuracy and precision data from QC samples (low, medium, high).

Parameter	Within-Run, n = 5			Between-Run, n = 10		
	Low	Medium	High	Low	Medium	High
Nominal concentration (ng·mL^−1^)	4.14	24.86	66.28	4.14	24.86	66.28
Mean found concentration (ng·mL^−1^)	4.60	23.68	62.19	4.62	23.01	59.98
RE (% Nom.)	111.15	95.25	93.83	111.53	92.56	90.49
RSD (%)	10.05	2.62	6.37	12.46	5.69	7.99

**Table 3:** Stability and recovery testing of serotonin in QC samples (low, medium, high).

	Nominal Concentration (ng·mL^−1^)	Freeze-Thaw Stability (3 Cycles)		Room Temperature Stability (24 h)		Recovery (%)
		Concentration Found (ng·mL^−1^)	Accuracy (%RE)	Concentration Found (ng·mL^−1^)	Accuracy (%RE)	
Low	4.14	4.46	7.73	4.27	3.14	90.00
Medium	24.86	25.85	3.99	23.79	−4.30	93.05
High	66.28	65.77	−0.76	60.75	−8.34	93.57
